# Model-Selection-Based Approach for Calculating Cellular Multiplicity of Infection during Virus Colonization of Multi-Cellular Hosts

**DOI:** 10.1371/journal.pone.0064657

**Published:** 2013-05-28

**Authors:** Mark P. Zwart, Nicolas Tromas, Santiago F. Elena

**Affiliations:** 1 Instituto de Biología Molecular y Celular de Plantas, Consejo Superior de Investigaciones Científicas – Universidad Politécnica de València, València, Spain; 2 The Santa Fe Institute, Santa Fe, New Mexico, United States of America; University of California, Riverside, United States of America

## Abstract

The cellular multiplicity of infection (MOI) is a key parameter for describing the interactions between virions and cells, predicting the dynamics of mixed-genotype infections, and understanding virus evolution. Two recent studies have reported *in vivo* MOI estimates for *Tobacco mosaic virus* (TMV) and *Cauliflower mosaic virus* (CaMV), using sophisticated approaches to measure the distribution of two virus variants over host cells. Although the experimental approaches were similar, the studies employed different definitions of MOI and estimation methods. Here, new model-selection-based methods for calculating MOI were developed. Seven alternative models for predicting MOI were formulated that incorporate an increasing number of parameters. For both datasets the best-supported model included spatial segregation of virus variants over time, and to a lesser extent aggregation of virus-infected cells was also implicated. Three methods for MOI estimation were then compared: the two previously reported methods and the best-supported model. For CaMV data, all three methods gave comparable results. For TMV data, the previously reported methods both predicted low MOI values (range: 1.04–1.23) over time, whereas the best-supported model predicted a wider range of MOI values (range: 1.01–2.10) and an increase in MOI over time. Model selection can therefore identify suitable alternative MOI models and suggest key mechanisms affecting the frequency of coinfected cells. For the TMV data, this leads to appreciable differences in estimated MOI values.

## Introduction

The cellular multiplicity of infection (MOI), the number of virions effectively infecting a cell, is a key parameter for understanding the dynamics and evolution of virus populations. This number is highly relevant for virus evolution because: (i) MOI is a determinant of the amount of genetic drift at the cellular level and the distribution of different viral genotypes over cells, (ii) complementation, recombination or reassortment between different genotypes can only occur in mixed-genotype infected cells, whilst mixed-genotype infections can only occur if the MOI >1, (iii) MOI will be a determinant of the respective importance of different levels of selection in viral evolution, and (iv) for many viruses, defective interfering particles can be generated and maintained for substantial periods of time if MOI is high. Competition between virus genotypes occurs at the between-host, within-host, within-tissue and within-cell levels, and the relative importance of different levels of selection is modulated by MOI. For example, low MOI levels (MOI ≤1) relax selection at the within-cell level and increase selection at higher levels [1]–[6]. Cellular MOI is therefore not only relevant to mechanisms at the cellular level, but is of great relevance to understanding viral evolution.

Although the importance of MOI is widely recognized, few estimates of the MOI of a virus in a complex multi-cellular host have been made. Three experimental approaches have been used. First, the rate at which a non-infectious virus is lost from a mixed-genotype population allows for estimation of MOI [7], leading to an estimated MOI of 4.3 during the final round of baculovirus replication in an insect host [8]. Second, a more direct approach to measure MOI is to infect a host with two marked virus variants and subsequently identify which variants are present in individual cells using sophisticated fluorescent-marker-based [2], [9] or PCR-based methods [10]. MOI is then estimated by using a simple mathematical model that considers how many cells have been infected by one or both variants. Using this approach, it was shown that during *Tobacco mosaic virus* (TMV) infection of *Nicotiana benthamiana* there are few cells coinfected by both virus variants, suggesting MOI is low and does not increase above 2 [9], [11]. For *Cauliflower mosaic virus* (CaMV) infection of *Brassica rapa* MOI rose from 2 to 13 as the virus expanded, and dropped to 2 again as the infection progressed even further [10]. Finally, tracking of infection during cell-to-cell expansion of two virus variants was used to estimate MOI during primary infection of *Soil-borne wheat mosaic virus* (SBWMV), rendering an estimated MOI of 6 and 5 for the first and second rounds of cellular replication in the inoculated leaf, respectively [2]. This elegant approach renders estimates of cellular MOI during the first few rounds of cellular infection, but more general application thereof may be difficult.

At first glance the concept of MOI is straightforward: it is the mean number of virions successfully infecting a population of cells. There are, however, two possible ways to define MOI: (i) the number of infecting virions over the total number of cells, which is referred to as *m_T_* and has a range [0,∞), or (ii) the number of infecting virions in infected cells, *m_I_*, and has a range [1,∞). Both definitions are valid and are likely to be used in different contexts. The first definition is particularly useful for describing manipulable units in an experiment (e.g., virion dose and the number of cells, for infection of cultured cells). The second definition, however, gives a more readily interpretable value for understanding the population genetics and evolution of a virus population. In this case only infected cells are of interest because no viral replication or interactions between genotypes occur in uninfected cells. However, in both cases there are problems when applying these concepts to a complex multi-cellular host. In particular, two important assumptions are being made: (i) the population of cells is homogenous, with each cell being equally susceptible to viral infection, and (ii) there is free mixing of virions and cells, such that each cell is equally accessible to virions. Moreover, if two virus variants are used to estimate MOI, then there must also be free mixing of the two virus variants. Although these assumptions may be largely met for a monolayer of cultured cells, they will probably not be met for a multi-cellular organism, with its complex spatial organization of differentiated cells with varying susceptibilities [12], [13]. A key question is therefore to what extent the assumptions of current MOI models are met, and whether this has important implications for making meaningful *in vivo* MOI estimates.

Two mathematical models for estimating MOI, based on the infection of a host with two marked virus variants and subsequently the identification of which variants are present in individual cells, have been proposed by González-Jara et al. [9] and Gutiérrez et al. [10]. In this paper, these studies will henceforth be referred to as Study 1 [9] and Study 2 [10]. Given that it represents a fundamentally different approach to estimating MOI, the model and data presented in Miyashita & Kishino [2] will not be considered here. Our aim was to develop a better, model-selection-based method to estimate MOI in complex multi-cellular organisms in which simple models are likely to give aberrant estimates of MOI. A new approach and methods for calculating MOI for virus colonization of multi-cellular hosts are presented, and consideration is given to the implications of using different MOI estimation methods. We test whether the assumption of a Poisson-distributed number of infecting virions over cells is warranted, seven new models for estimating MOI are developed, and it is shown how model selection can be applied. Finally, MOI estimates generated by different methods for the two experimental datasets are compared. These results reveal that reported methods for estimating MOI are satisfactory for CaMV, whereas for TMV an alternative model is better supported and leads to different MOI estimates.

## Materials and Methods

First, a description of the models used to estimate MOI in Studies 1 and 2 is provided, along with some minor modifications to the model fitting procedure for Study 1. Subsequently, a method for determining whether the data follow a Poisson distribution is presented. Next, alternative models for MOI estimation are developed, and the model fitting and selection procedure used is described.

### Description of Previously Reported Approaches to MOI Estimation

#### Model 1: MOI estimation method of Study 1

In order to estimate MOI, González-Jara et al. [9] proposed that *m_I_* (the number of infecting virions, considered only over the virus-infected cells) be seen as a constant, and that the proportion of cells infected only by variant *A* is then the zero-term of a binomial distribution of the number of infecting virions of variant *B*:
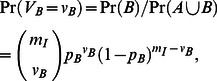
(1)where *V_B_* is a random variable describing the number of virions of variant *B* that is infecting a virus-infected cell, *v_B_* is a realization of *V_B_,* and *p_B_* is the frequency of variant *B* estimated from the data as:



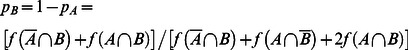
(2)Throughout this study, we use *f*(·) *to* denote the observed frequencies of each class of infected cells, whereas Pr(·) denote the expected probabilities thereof. In equation 1, a statement of equivalence to 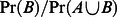 is included to stress that the binomial probability is calculated only over the fraction of infected cells, 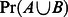. Given that *m_I_* is constant, the expected frequency of mixed-variant infected cells in the fraction of infected is then:
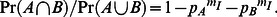
(3)


Note that in this computation the observed frequency of uninfected cells 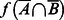 is not taken into consideration. The predicted frequency of single and mixed-variant infected cells can then be compared to the observed frequency by means of the multinomial likelihood, although in Study 1 a *G* test [14] was used. Nevertheless, to be able to compare the different methods for MOI estimation, one can simply compare the observed fraction of coinfected cells, 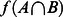, to model predictions by means of the binomial likelihood. For each individual observation:
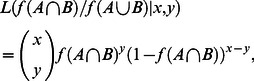
(4)where *x* is the total number of infected cells and *y* is the number of mixed-variant infected cells, and we consider the sum of log-likelihoods as a measure of model fit.

#### Model 2: MOI estimation method of Study 2

**Figure 1:**
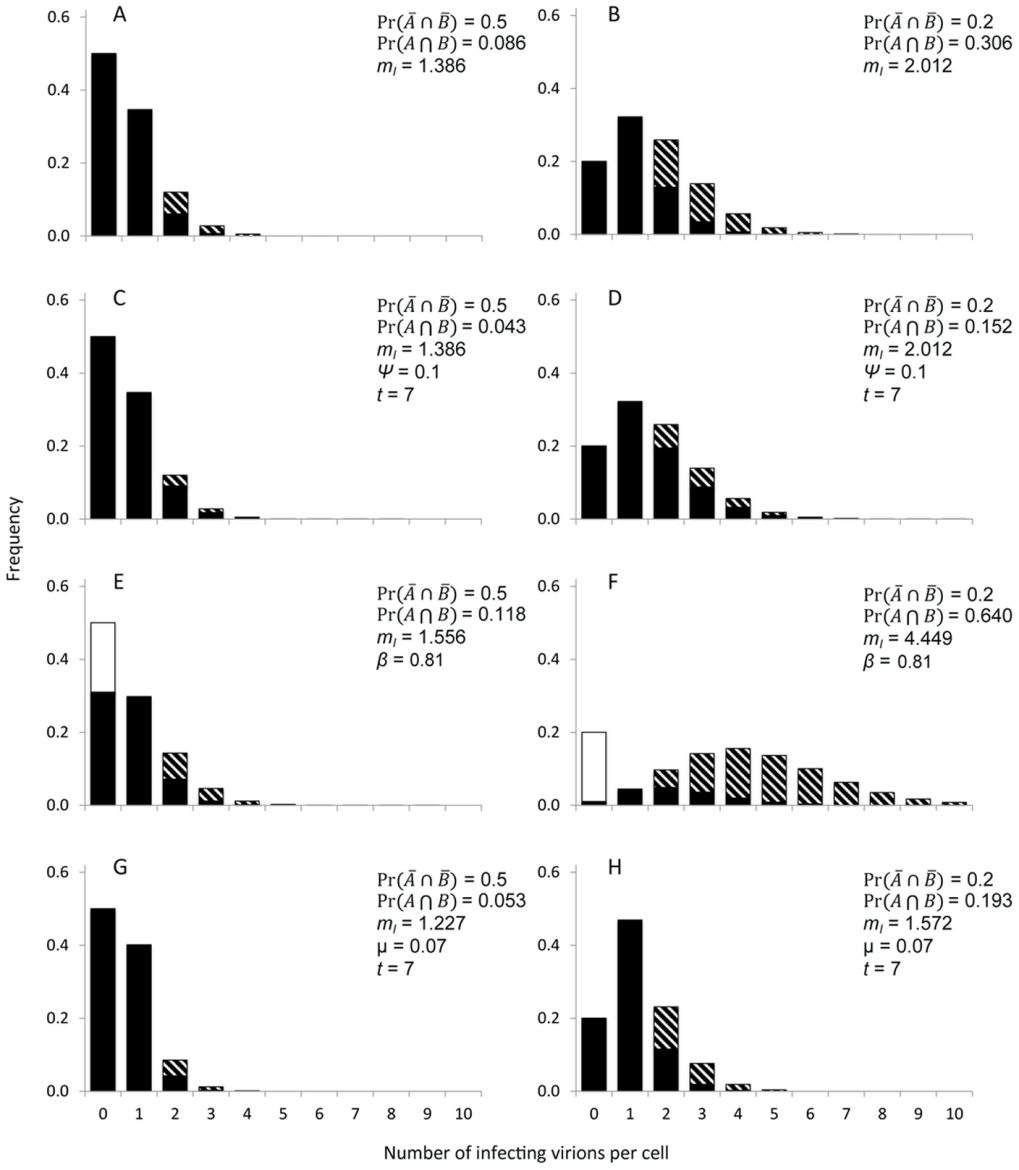
MOI Models. This figure illustrates the different MOI models. For all panels, the number of infecting virions per cell is on the abscissae, and the frequency thereof is on the ordinate. The black portion of bars is the frequency of single-variant infected cells, whereas the striped portion corresponds to the frequency of mixed-variant infected cells. The white portion of bars in Panels E and F corresponds to cells that are not infected by the virus because they are invulnerable to infection, as a consequence of the aggregation of virus-infected cells. For all left-hand panels, half of the cells are uninfected (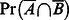 = 0.5), whereas for the right hand panel, only one-fifth of the cells remain uninfected (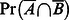 = 0.2). For each panel we also report the overall frequency of mixed-variant infections (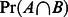), the mean number of infecting virions in infected cells (*m_I_*), and model parameters. The frequency of the two virus variants is assumed to be 1∶1 in all cases. Panels A and B illustrate Model 2, the simple Poisson model. Panels C and D illustrate Model 3, which incorporates the effects of spatial segregation of virus variants during expansion, the strength of which is determined by time *(t*) and a constant *Ψ*. Note that *m_I_* and the overall shape of the distributions are the same; the only difference is the lower frequency of mixed-variant infections for Model 3. Panels E and F illustrate Model 4, which incorporates a fraction of cells *β* that can become infected, and a fraction 1 − *β* that cannot. For this model, the zero-term of the Poisson distribution is composed of only those cells can become infected but are in fact uninfected, leading to a higher *m_I_* and 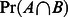. Panels G and H illustrate Model 5, which incorporates super-infection exclusion as determined by time and a parameter *μ*. This leads to a reduction of both *m_I_* and 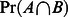. For Model 5, we have not illustrated *ω*, the level of super-infection exclusion at *t = *0, which has the same effect as *μ* but in a time independent manner.



Study 2 [10] employs broadly the same experimental approach as Study 1, although the authors’ method for MOI estimation assumes that the distribution of infecting virions per cell follows a Poisson distribution rather than being constant (Figures 1A and B), such that 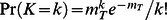. The frequency of mixed-variant infected cells is then:

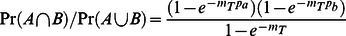
(5)See [15] for a detailed derivation of this equation. Here, *p_A_* could be derived from the same data with equation 2, although in the study the authors obtain estimates of *p_A_* by determining the frequencies of variants in the whole leaf by means of qPCR [10]. The authors minimize the negative log likelihood, by obtaining binomial likelihoods from the comparison of predicted and observed number of mixed-variant infected cells, as in equation 4.

### Testing Whether the Distribution of Infecting Virions Follows a Poisson Distribution

If the number of infecting virions over the total number of cells (*m_T_*) follows a Poisson distribution, then the observed fraction of uninfected cells 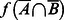 can be used to predict *m_T_* [15], the relationship being:
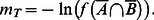
(6)


This relationship is an inevitable outcome of the assumption that *m_T_* follows a Poisson distribution, which does not seem to be a contested assumption [10]. One can partition *m_T_* over the two variants *A* and *B* using equation 2, and predict the frequency of mixed-variant infections using equation 5. The predicted and observed values for the frequency of mixed-variant infected cells can then be compared using a one-sided exact binomial test. This test was performed for the reported experimental data of Studies 1 and 2, pooling the data from multiple replicates (i.e., different plants). Data were pooled because of two characteristics of Study 2: (i) the PCR method, although having many advantages, entails that the number of replicate cells tested per plant (≤50) is limited and will introduce more sampling error than the fluorescence-based method, and (ii) the test proposed here can only be applied when there is a fraction of uninfected cells, which due to the high cellular infection rate and the limited number of cells tested is not always the case for individual plants in Study 2.

### Alternative MOI Models

A series of alternative models of MOI, which incorporate mechanisms that could occur during viral infection of a complex multi-cellular host, were formulated.

#### Model 3

Models 1 or 2 may fail due to spatial segregation of virus variants during expansion within the host plant [2], [16], [17]. Spatial segregation is understood to be the fact that the two variants occupy different spatial locations within the plant, and that it is therefore impossible for cellular coinfection to occur in some locations in the host, irrespective of the actual MOI. Two assumptions are made to model this process: (i) the fraction of cells in which both variants are present (i.e., both virus variants have a local presence) decreases at a constant rate, and (ii) once the variants have been segregated, the rate at which they reunite is so low that unification can be ignored. Under these assumptions, the fraction of mixed-variant infections will be smaller than expected for a given value of *m_T_*, such that:
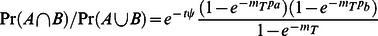
(7)where *ψ* is a constant determining how strong the effects of spatial segregation are and *t* is time, as measured in days post-inoculation (dpi). The range of *ψ* is therefore [0,∞); negative values are not possible as spatial segregation cannot increase the frequency of mixed-variant infections. Note equation 6 can be used to predict the MOI with this model; *ψ* is only estimated for model selection and does not affect MOI. As *ψ* increases, so does the effect of spatial segregation of variants over time, although the distribution of infecting virions per cell is not changed for this model (Figures 1C and D) with respect to Model 2.

This model of spatial segregation captures the physical process that results in a given fraction of mixed-variant infected cells at a given time point, without mechanistically modeling the reasons why this spatial segregation has occurred. Possible reasons why spatial segregation occurs include population bottlenecks during the colonization of new organs or tissues, host spatial structure and super-infection exclusion (Model 5). The key point is that the distribution of virions remains unchanged, whereas for any given number of infecting virions the probability of both variants being present drops to zero for a part of the population of cells. Consequently, a plausible and simple approach to modeling the dynamics of spatial segregation will be an exponential function, but other mathematical functions could be considered as well without having major effects on model behavior.

#### Model 4

For viruses in general, the probability of infection per virion may not be the same over all cells. Not all cells may be equally vulnerable to viral infection [12], [13], [18] due to differences in (i) the probability of infection even if a cell is exposed to the virus, (ii) the probability that a cell will be exposed to the virus, or (iii) both. This situation is exacerbated in plant viruses, because they spread locally by means of cell-to-cell movement [2], [19]. Cells can only be infected by cell-to-cell movement if they are adjacent to a virus-infected cell, which results in the spatial aggregation of virus-infected cells [2], [16]. If the probability of infection varies over cells – for whatever reason – such differences will irrevocably result in a higher frequency of mixed-variant infected cells at a given level of cellular infection [20]. We chose to model these processes using an approach developed by Barlow [21], [22], such that:
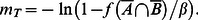
(8)
*β* has a range [0,*β_max_*), where *β_max_* is the smallest value of 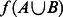 for the dataset to which the model is to be fitted. There are two ways to interpret the parameter *β*. First, there are only two unconnected patches of cells, and the one containing no infected cells (and therefore no infectious cells). The cells in the uninfected patch can then be seen as being invulnerable to infection, and over the whole population of cells there is a fraction 1– *β* that is therefore invulnerable to infection. Second, *β* can be seen as a measure of the spatial aggregation of virus-infected cells. For the latter, when *β = *1 there is no aggregation of infected cells and when *β* ≈ 0 there is maximum spatial aggregation of infected cells. Because our modeling here concerns plant viruses in which cell-to-cell movement is known to play an important role, the most reasonable interpretation of *β* is the spatial aggregation of virus-infected cells. On other hand, to illustrate the model it is easiest to consider the effects of a predicted fraction of invulnerable cells has on MOI and mixed-variant infections (Figures 1E and 1F). As the fraction of invulnerable cells increases, so does *m_T_*.

#### Model 5

The assumption of independent action of viruses during infection might fail. Plant viruses are thought to in some cases exclude each other at the cellular level [16], a phenomenon known as super-infection exclusion. In this case, the actual MOI would be lower than predicted by the Poisson model. Moreover, to allow for the possibility that such effects may change in strength during the course of infection, the model was formulated as:
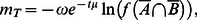
(9)where *ω* is a constant that determines the strength of super-infection exclusion at the cellular level at *t = *0, and *μ* is a constant that determines how super-infection exclusion changes over time. Given that we are not aware of a molecular mechanism that would have the opposite effect of exclusion (i.e., inclusion of virions in a cell that has already been infected by one virion), we set the range of *ω* to [0,1] and *μ* to [0,∞). The key point of the model is that the distribution of infecting virions is affected, whilst for any number of infecting virions the probability of both variants occurring is the same as Model 2 (Figure 1G and H).

Super-infection exclusion will lead to a reduction of mixed-variant infections observed for each time point, similar to spatial segregation of variants (Model 3). However, unlike spatial segregation, super-infection exclusion leads to a reduction in mixed-variant infections because it affects the distribution of infecting virions, lowering its mean (Figures 1G and 1H). It is important to note that super-infection exclusion may also lead to spatial segregation over time. If such an effect were to occur, one would expect a lowered frequency of mixed-variant infections at every time point, and the segregation of variants over time. Model selection would then be expected to identify Model 8 (see below) as the best supported model, because it incorporates both super-infection exclusion and spatial segregation of variants, while not specifying the mechanism that results in spatial segregation of variants.

To allow for dynamic change in the strength of super-infection exclusion, the mean number of infecting virions was modulated with an exponential function. It could not be predicted *a priori* how super-infection exclusion might change dynamically. An exponential function and an extra constant regulating the strength of super-infection exclusion at *t = *0 (*ω*) were therefore incorporated because it offers greater flexibility than a linear model and first attempts to fit the model showed this function allowed for better fit than alternative functions. As its effects on mixed-variant infections are similar, Model 5 is in effect a ‘stalking horse’ for Model 3.

#### Models 6, 7, 8 and 9

The different alternative models of MOI were also combined, especially since a clear hypothesis could not be formulated *a priori* on what effect or combinations of effects may account for the discrepancies between the Poisson model and the data. The combined models and their free parameters are given in Table 1. For Models 6, 8 and 9, *ψ* does not affect MOI estimates directly but this parameter could affect estimates of other model parameters (*β*, *ω* and *μ*) that do directly affect MOI during model fitting.

**Table 1:** Overview of Models 2 through 9.

Model	Spatial segregation	Aggregation of virus-infected cells	Super-infection exclusion	Model parameters
2				–
3	X			*Ψ*
4		X		*β*
5			X	*ω, μ*
6	X	X		*Ψ, β*
7		X	X	*β, ω, μ*
8	X		X	*Ψ, ω, μ*
9	X	X	X	*Ψ, β, ω, μ*

An X indicates the mechanisms incorporated by the different models. Note that Model 2 incorporates none of these mechanisms, and that Models 3–5 incorporate only one mechanism. Model 1 is not included in the overview, given that we can only make a formal comparison of the Poisson-based models.

### Model Selection and MOI Predictions

MOI models 2 through 9 all link 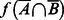 to 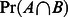, allowing one to perform model selection to choose the model best supported the data. Model 2 does not have any parameters that need to be estimated. Models 3 through 9 were fitted to the pooled data sets using the statistical computing software R 2.14 [23]. We first performed grid searches, which minimized negative log likelihood (NLL; determined using equation 4), over large parameter spaces to search for a global solution. Stochastic hill climbing was then performed separately on 1000 bootstraps of the data, rendering parameter estimates and their 95% confidence interval (CI). The Akaike Information Criterion (AIC) was used to determine how much support the data provide to a particular model. The estimates of *m_T_* rendered by the fitted models are then the predictions of MOI, which were subsequently used to estimate *m_I_*. Assuming both *m_I_* and *m_T_* are Poisson distributed, the relationship between *m_I_* and *m_T_* will be that same as that of zero-truncated Poisson distribution and a complete Poisson distribution [24]:
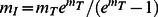
(10)


Bootstrapping was also used to obtain the 95% CI for MOI estimates.

### Fitting of the Logistic Growth Model to the Data

In order to estimate the fraction of cells which will eventually become infected, the logistic growth model:
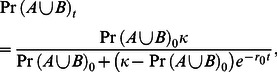
(11)was fitted to the inoculated leaf data of Study 1 and the complete data of Study 2 using nonlinear regression (SPSS 20.0), where *κ* is the carrying capacity and *r*_0_ is the initial growth rate.

## Results

### The Two Methods for Estimating MOI Render a Different Parameter

**Figure 2:**
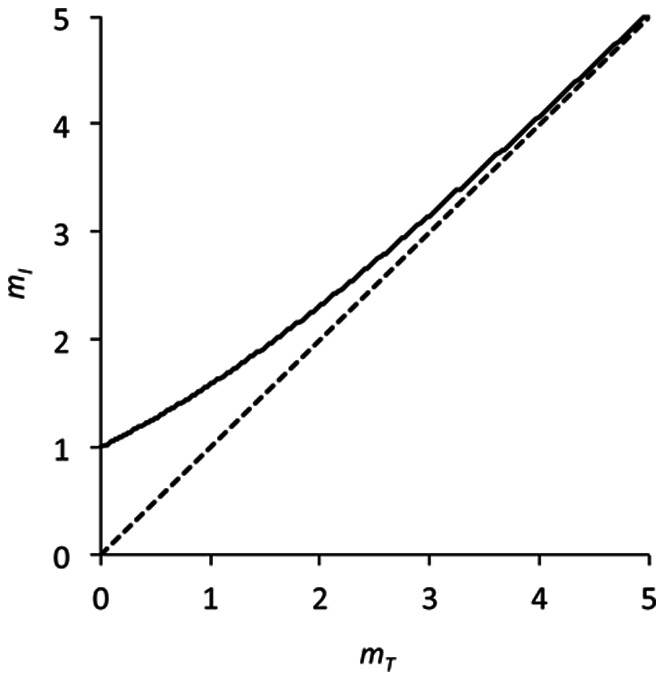
A comparison of *m_T_* and *m_I._* The relationship between *m_T_* (abscissae) and *m_I_* (ordinate) is plotted as the continuous line. The dotted line is a 1∶1 relationship, given for comparative purposes. *m_I_*>*m_T_*, although for higher values (>4) the difference becomes very small. Note that *m_T_* and has a range [0,∞) whilst *m_I_* has a range [1,∞).

The MOI estimation methods of Studies 1 [9] and 2 [10] provided different parameters, although both are called MOI. The method of Study 1 (Model 1 in our study) estimates *m_I_*, the MOI over infected cells, given that only this fraction is considered (equation 3). For the method of Study 2 (Model 2 in our study), the expected frequency of mixed-variant infected cells is divided by the fraction of uninfected cells, and hence *m*-values concern the mean of a non-truncated Poisson distribution (equation 5). This difference is non-trivial; *m_I_* and *m_T_* have a different range, and the relationship between their respective means is given by equation 10. For low levels of infection, *m_I_*>*m_T_* whereas for higher values *m_I_* ≈ *m_T_* (Figure 2). Nevertheless, in this case the estimates reported in the two studies are roughly comparable if considered as *m_I_* estimates: for Study 2 the MOI is high, the majority of cells tend to be infected [10] and hence the zero fraction of the predicted Poisson distribution is small and does not lower the estimate much.

### The Simple Poisson Model is not Supported for either Data Set

The two previously reported methods for calculating MOI differ with respect to whether the number of infecting virions is assumed to be constant (Model 1) or variable (Model 2), following a Poisson distribution. A Poisson distribution represents the minimal variation that would be expected for independently acting virions infecting cells, and as such we would expect *a priori* that this is a significant improvement. However, if the assumptions underlying the Poisson-based model are not met, there could be important implications for MOI estimates. A simple test of whether the experimentally observed distribution of the two virus variants over cells is similar to that predicted by a Poisson distribution was therefore developed (See Materials and Methods), and the Poisson model was rejected for both datasets (Table 2). Note that a similar test could not be performed for Model 1, since this model is concerned only with the fraction of infected cells.

**Table 2:** Test of the Poisson model.

Study	Leaf	Day	*p_a_*	*m_T_*	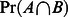	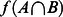	Binomial *P*	Δ^a^
1	I^b^	2	0.505	0.03	0.007	0.030	0.080	
	I^b^	4	0.447	0.14	0.034	0.039	0.524	
	I^b^	7	0.431	0.74	0.181	0.045	<0.001***	<
	I^b^	11	0.375	1.72	0.380	0.027	<0.001***	<
	S^c^	4	0.117	1.06	0.109	0.040	<0.001***	<
	S^c^	7	0.136	1.08	0.126	0.051	<0.001***	<
	S^c^	11	0.303	1.73	0.348	0.030	<0.001***	<
2	6	15	0.898^d^	3.40	0.289	0.207	0.019*	<
	12	27	0.866^d^	2.21	0.246	0.426	<0.001***	>
	21	41	0.924^d^	5.12	0.321	0.476	<0.001***	>
	33	56	0.788^d^	3.78	0.536	0.430	0.006**	<
	43	72	0.823^d^	3.80	0.478	0.223	<0.001***	<

A test of the Poisson model, using the proportion of uninfected cells to predict the occurrence of mixed-variant infected cells.

a Δ indicates whether the observed frequency of mixed-variant infected cells 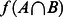 is greater than or less than the predicted value 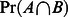, if the difference is significant.

b The inoculated leaf.

c Systemically infected leaf.

d In these cases *p_a_* is the mean qPCR-measured frequency, instead of being derived from the frequencies of infected cells, given that these data are not reported in the study.

For the Study 2 data, the Poisson model was rejected in all five cases, and the observed frequency of mixed-variant infections was significantly lower than model predictions in three cases and significantly higher in two cases. To determine if this is not an effect of pooling the data, tests were also performed for data from individual plants when possible (i.e., 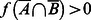). Despite the decrease in power due to the smaller numbers, the Poisson model is still rejected in six out of 14 cases: four cases being significantly lower and two cases significantly higher (data not shown). Although the differences between the data and model are highly significant for Study 2, they are not as drastic as for the data of Study 1.

### Model Selection Results

A set of probabilistic models for predicting MOI, incorporating different mechanisms that may account for the rejection of Model 2, were developed. For a detailed description of these seven models (Models 3 to 9), see the Materials and Methods section. An overview of the models is given in Table 1, and a description of the models incorporating a single additional mechanism (Models 3, 4 and 5) is given in Figure 1. When model selection was performed over the set of eight models (Model 2 as the null model, and Models 3 to 9), it was found that for Study 1 Model 3 had the most support (Table 3). Model 3 incorporates only a single additional mechanism: spatial segregation of variants over time. There was also some support for Model 6 (Table 3), which incorporates both spatial segregation of variants and aggregation of infected cells. However, the difference in model fit (i.e., NLL) between Models 6 and 3 is minimal, suggesting that the most important mechanism required is the spatial segregation of virus variants. Similar results were obtained for the data of Study 2 (Table 4). Overall, the best-supported model was Model 6, but of the models incorporating only a single additional mechanism, Model 3 was again the best supported. Moreover, given that most cells are virus-infected in the data of Study 2 [10], aggregation of infected cells cannot play a very important role. Therefore, for both data sets the spatial aggregation of variants best describes the discrepancies between the data and the simple Poisson model, with a secondary role for the aggregation of virus-infected cells in Study 2. However, model parameter estimates reveal that both effects are considerably weaker for the data of Study 2, where the discrepancies between the data and the Poisson model are also smaller (Table 2).

**Table 3:** Model selection with the data of Study 1.

Model	Parameter estimates	NLL	AIC	ΔAIC	AW
2	–	2142.528	4285.056	4179.399	0
3	*Ψ = *0.213 [0.200–0.224]	51.829	105.657	–	0.595
4	*β = *1 [0.976–1]	2142.528	4287.056	4181.399	0
5	*ω = *1 [0.998–1]	53.400	110.800	5.142	0.046
	*μ = *0.217 [0.205–0.228]				
6	*Ψ = *0.218 [0.206–0.235]	51.777	107.554	1.896	0.231
	*β = *0.979 [0.931–1]				
7	*β = *1 [0.970–1]	53.400	110.800	7.142	0.017
	*ω = *1 [*]				
	*μ = *0.217 [0.206–0.230]				
8	*Ψ* = 0.213 [0.099–0.219]	51.829	109.657	4.000	0.081
	*ω = *1 [*]				
	*μ = *0 [0–0.116]				
9	*Ψ = *0.218 [0.100–0.229]	51.777	111.554	5.896	0.031
	*β = *0.979 [0.939–1]				
	*ω = *1 [*]				
	*μ = *0 [0–0.120]				

MOI Models 2–9 were fitted to the pooled data of Study 1 [9]. We give estimates of model parameters with the 95% CI in parenthesis, and an asterisk indicates the lower and upper 95% CI limits coincide with the estimate parameter value. For each model we also provide the negative log likelihood (NLL), Akaike information criterion (AIC), the difference between a given model and the best-supported model in AIC (ΔAIC), and the Akaike Weight (AW). Overall, Model 3 is the best-supported model, although there is also some support for Model 6, which combines the single mechanisms incorporated in Models 3 and 4. The improvement in model fit (NLL) between Models 6 and 3 is, however, minimal.

**Table 4:** Model selection with the data of Study 2.

Model	Parameter estimates	NLL	AIC	ΔAIC	AW
2	–	65.029	132.057	33.497	0
3	*Ψ = *0.004 [0.001–0.024]	55.579	113.158	14.597	0.001
4	*β = *1 [0.995–1]	65.029	134.057	35.497	0
5	*ω = *1 [*]	56.540	117.079	18.519	0
	*μ = *0.005 [0.002–0.009]				
6	*Ψ = *0.005 [0.002–0.008]	47.280	98.561	–	0.755
	*β = *0.995 [0.995–1]				
7	*β = *0.995 [0.995–1]	47.955	101.910	3.349	0.142
	*ω = *1 [0.902–1]				
	*μ = *0.006 [0.003–0.098]				
8	*Ψ* = 0.004 [0–0.019]	55.579	117.158	18.597	0
	*ω = *1 [0.986–1]				
	*μ = *0 [0–0.006]				
9	*Ψ = *0.005 [0–0.009]	47.280	102.561	4.000	0.102
	*β = *0.995 [0.995–1]				
	*ω = *1 [0.998–1]				
	*μ = *0 [0–0.008]				

MOI Models 2–9 were fitted to the pooled data of Study 2 [10]. We give estimates of model parameters with the 95% CI in parenthesis, and an asterisk indicates the lower and upper 95% CI limits coincide with the estimate parameter value. For each model we also provide the negative log likelihood (NLL), Akaike information criterion (AIC), the difference between a given model and the best-supported model in AIC (ΔAIC), and the Akaike Weight (AW). Overall, the best-supported model is Model 6, which combines the single mechanisms incorporated in Models 3 and 4. Of the models adding only one addition mechanism to the original Poisson model (Models 2–4), Model 3 leads to the greatest improvement in fit (i.e., it has the lowest NLL).

A logistic growth model was fitted to the data to estimate the carrying capacity (*κ*), expressed as a proportion of total cells, and test whether the kinetics of replication suggest that infection will saturate before all cells are infected. This procedure generated *κ* estimates, with the 95% CI in brackets, of 0.846 (0.756–0.936) for Study 1 and 0.961 (0.850–1.072) for Study 2. For Study 1 virus expansion appears stop to before all cells have become infected, whereas for Study 2, infection levels are very high and almost all cells become infected. If we consider the alternative interpretation of *β* as the frequency of cells which are vulnerable to infection, *κ* estimates from the logistic growth model should be roughly comparable to *β*. For Study 1, *β* estimates, with the 95% CI in brackets are 0.979 (0.931–1), whereas for Study 2 *β* is 0.995 (0.995–1) (Tables 3 and 4). The 95% CIs for the two estimated parameters therefore overlap for both Studies 1 and 2. Nevertheless, *κ* estimates appear to be somewhat lower than those of *β*, and the parameter estimates may not allow for a meaningful comparison as the maximum value of *β* is 1. Note that although *β* ≈ 1, for Study 2 it still has an effect on model fit and MOI estimates because most cells are infected (equation 8).

### Predicted MOI Values

**Figure 3:**
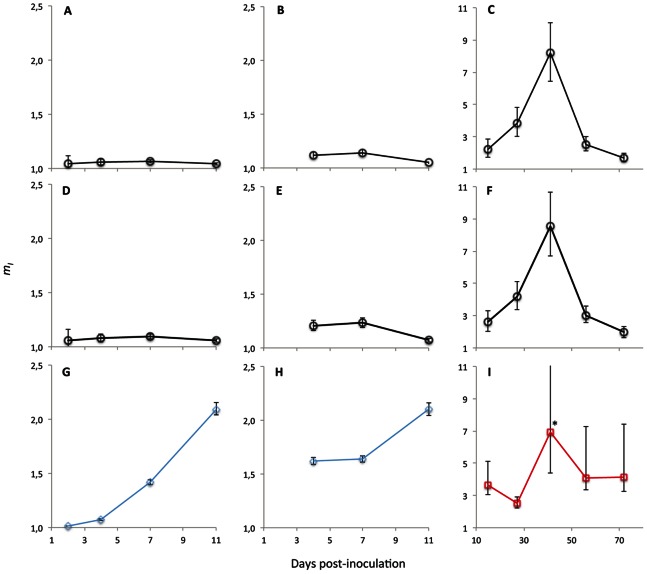
A comparison of *m_I_* estimates from Models 1, 2 and 6. The estimated MOI (*m_i_*) is given for the inoculated leaf in Study 1 (Panels A, D and G), for the systemic leaf in Study 1 (Panel B, E and H), and for different systemic leaves collected at different times points in Study 2 (Panel C, F and I) using Model 1 (Panels A–C), Model 2 (Panels D–F), Model 3 (Panels G and H, blue lines and diamonds) and Model 6 (Panel I, red lines and squares). Model 3 is the best-supported model for the Study 1 data, whereas Model 6 is the best-supported model for the Study 2 data. The days post-inoculation (dpi) are given on the abscissae, whereas *m_I_* is the ordinates. Error bars represent the 95% CI, and are marked with an asterisk when they extend to infinity (Panel I at 21 dpi). For the data of Study 1 (Panels A, B, D, E, G, and H), Models 1 and 2 both predict that MOI remains low throughout infection. On the other hand, Model 3 predicts that MOI increases over time, as this model incorporates the effects of spatial segregation of variants (Panels G and H). Note that Model 6 predictions are nearly identical to Model 3 predictions for Study 1. For the data of Study 2 (Panels C, F and I), model predictions are roughly similar and the dynamic pattern is the same. However, the differences in MOI over time are less pronounced for Model 6, in particular the decrease of MOI towards the end of infection. This difference is again due to predicted segregation of variants incorporated in Model 6, although the predicted effects thereof are much weaker for the data in Study 2 than in Study 1 (Tables 3 and 4).

To better understand the implications of the different models for MOI estimation, three approaches were used to estimate MOI for data from Studies 1 and 2. First, the method of Study 1 (Model 1) was used to estimate *m_T_*. Second, the method of Study 2 (Model 2) was used to estimate *m_T_*, and equation 10 was then used to estimate *m_I_* values. Third, the best-supported model was used to estimate *m_I_*, by means of equations 8 and 11. For Study 1, estimated *m_I_* values were highly similar for Models 1 and 2, whilst Model 3 gave a diverging result (Figure 3). The results for Models 1 and 2 are similar to the results of Study 1 [9], [11] and the results for pooled data of Study 2 (see Figure S2 in [10]). The similarity of the results for Models 1 and 2 is somewhat surprising, given that Model 1 assumes the number of infecting virions to be fixed and Model 2 assumes it follows a Poisson distribution. Model 3 renders much higher estimates of MOI for the data of Study 1 than Models 1 and 2. This difference results from including the predicted effects of spatial segregation of variants as infection progresses, because this mechanism allows for the combination of a high rate of cellular infection and a low rate of mixed-variant infected cells. For the data of Study 2, MOI estimates of the different models diverge less, although Model 6 predicts that the MOI will remain somewhat more constant over time than do Models 1 and 2. Particularly at later time points in infection, Model 6 predicts MOI values that are considerably higher than those predicted by Models 1 and 2. This discrepancy can again be attributed to the spatial segregation of virus variants in Model 6.

## Discussion

MOI has been estimated for complex multi-cellular organisms using sophisticated experimental setups and mathematical models [8], [9], [10]. Nevertheless, the concept of MOI is still largely based on virus infections in cell culture, where there is a homogeneous population of cells in a largely unstructured environment in which virions can freely mix. By considering MOI during virus spread at the cellular level, Miyashita & Kishino [2] have developed the concept further. However, such a development is also necessary when considering MOI at the level of the whole host or host organs. By means of comparing the data to predictions of the Poisson null-model (Model 2) and by performing model selection over a range of alternative models (Models 3 to 9), it has been shown here that the simple ‘cell culture’ model is in some cases not sufficient to estimate MOI. Moreover, these results demonstrate that the alternative models can render better-supported estimates of MOI for some data sets. As such, the combination of these alternative models of MOI and model selection is a useful tool for calculating MOIs based on experimental data.

The new methods presented here, however, also have additional advantages. For both studies, similar models received the most support from the data. For the datasets of both studies, the best-supported model incorporated the spatial segregation of variants (Model 3). For Study 2, it was clear that a further mechanism was also required: the aggregation of virus-infected cells (Model 4), as embodied in the model combining these two mechanisms (Model 6). Model 6 also had limited support for the Study 1 data, but the improvement in fit over Model 3 is minimal. Knowing what mechanisms lead to the rejection of the Poisson model is of interest for better understanding the infection process. The fact that the including the spatial segregation of virus variants over time leads to high levels of support is therefore noteworthy and suggests that perhaps this model has a degree of generality. Nevertheless, the differences in support for the models (i.e., ΔAIC values in Tables 3 and 4) again suggest that the deviations from the simple Poisson model (Model 2) are much smaller for the data of Study 2 than of Study 1. Moreover, it should be considered that the ΔAIC between Models 3 and 5, which incorporates time-varying super-infection exclusion, was not very large for both datasets (Tables 3 and 4). Further experimental confirmation of these results is therefore needed before it can be concluded with a reasonable degree of certainty what is the chief mechanism leading to low levels of cellular co-infection.

The approach presented here does not lead to highly divergent results for the data of Study 2. There are some differences in MOI estimates, but the dynamic pattern is the same and values are roughly comparable (Figure 3). Moreover, although the results do not support a Poisson-distributed number of infecting virions, the discrepancies between the data and model are minor. Furthermore, during model selection the improvement in model fit – although being appreciable – is not nearly as large as for the Study 2 data (Tables 3 and 4). Finally, small values are estimated for *ψ* and *β*, suggesting that the deviation from the Poisson model is not important. Overall, therefore, our analysis suggests that for the estimation of MOI for the data of Study 2, Models 1 and 2 are satisfactory, given that the deviations from the Poisson model are small and do not have large effects on estimates. It can therefore be concluded that even if the data only roughly approximate the assumption of a Poisson-distributed number of infecting virions, Models 1 and 2 still give surprisingly good estimates of MOI. A test of whether the data meet this assumption (e.g., Table 2) is therefore a useful diagnostic tool, although given the apparent robustness of Models 1 and 2, considering whether the expected frequencies are ‘ball park’ estimates is more important than statistical significance. These results therefore exemplify the limitations of simple models when model assumptions are not met (results for Study 1), while simultaneously demonstrating that these same simple models are remarkably robust to small violations of model assumptions (results for Study 2).

For the data of Study 1, MOI values predicted by Model 3 are much higher than MOI values predicted by Models 1 and 2. In this case, model selection predicts that there will be strong spatial segregation of variants during TMV infection of *N. benthamiana*. Whether these predicted patterns occur during actual infection of plants can be empirically tested, and suggests new avenues of experimental research. Ultimately, our research strongly reinforces the idea that for the estimation of *in vivo* MOI in multicellular organisms, it is indispensable to move beyond the ‘cell culture’ conceptual model and consider the effects of spatial processes occurring during viral expansion in these complex hosts [2].
